# Hyponatremia in Patients with Cirrhosis of the Liver

**DOI:** 10.3390/jcm4010085

**Published:** 2014-12-31

**Authors:** Mauro Bernardi, Carmen Serena Ricci, Luca Santi

**Affiliations:** 1 Department of Clinical and Surgical Sciences, Alma Mater Studiorum—University of Bologna, 40139 Bologna, Italy; E-Mails: carmenserena.ricci@hotmail.it (C.S.R.); lucasanti@hotmail.it (L.S.); 2 Semeiotica Medica, Policlinico S. Orosla-Malpighi, Via Albertoni, 15, 40138 Bologna, Italy

**Keywords:** hyponatremia, liver cirrhosis, hepatic encephalopathy, liver transplantation, Vaptans

## Abstract

Hyponatremia is common in cirrhosis. It mostly occurs in an advanced stage of the disease and is associated with complications and increased mortality. Either hypovolemic or, more commonly, hypervolemic hyponatremia can be seen in cirrhosis. Impaired renal sodium handling due to renal hypoperfusion and increased arginine-vasopressin secretion secondary to reduced effective volemia due to peripheral arterial vasodilation represent the main mechanisms leading to dilutional hyponatremia in this setting. Patients with cirrhosis usually develop slowly progressing hyponatremia. In different clinical contexts, it is associated with neurological manifestations due to increased brain water content, where the intensity is often magnified by concomitant hyperammonemia leading to hepatic encephalopathy. Severe hyponatremia requiring hypertonic saline infusion is rare in cirrhosis. The management of asymptomatic or mildly symptomatic hyponatremia mainly rely on the identification and treatment of precipitating factors. However, sustained resolution of hyponatremia is often difficult to achieve. V2 receptor blockade by Vaptans is certainly effective, but their long-term safety, especially when associated to diuretics given to control ascites, has not been established as yet. As in other conditions, a rapid correction of long-standing hyponatremia can lead to irreversible brain damage. The liver transplant setting represents a condition at high risk for the occurrence of such complications.

## 1. Epidemiology and Prognostic Importance of Hyponatremia in Cirrhosis

Reduced serum sodium concentration is a common finding in patients with cirrhosis [1,2], being the most common electrolyte disorder in this setting. Indeed, about 20% of patients have values lower than 130 mmol/L, which is the current definition of hyponatremia in cirrhosis [3]. However, even though patients with cirrhosis and serum sodium concentration between 130 and the lower normal limit of 135 mmol/L could not be considered as hyponatremic according to this definition, they present pathogenic and clinical features similar to those with serum sodium lower than 130 mmol/L. With the cutoff of 135 mmol/L, the prevalence of hyponatremia rises to almost 50%. Instead, the occurrence of severe hyponatremia, that is serum sodium concentration lower than 126 mmol/L, is rare and its prevalence is 6% [2].

Although hyponatremia can be found in patients with early or moderately advanced cirrhosis belonging to classes A and B of Child-Pugh classification [4], in most cases it occurs in an advanced disease (Child-Pugh class C). The relationship between hyponatremia and severity of cirrhosis is further evidenced by its close association with the occurrence of complications: indeed, the prevalence of hepatic encephalopathy, hepatorenal syndrome and spontaneous bacterial peritonitis is substantially higher in patients with serum sodium concentration ≤130 mmol/L than in those with higher levels. Moreover, among patients with ascites, those with hyponatremia have a lower response to diuretics, a higher incidence of refractory ascites, and more often need therapeutic paracentesis at shorter intervals [2].

As reported above, severe hyponatremia, that would require immediate and specific treatment, is relatively rare in cirrhosis. Therefore, the occurrence of mild to moderate hyponatremia has mainly to be appraised for its clinical meaning. In fact, the occurrence of hyponatremia represents an independent outcome predictor for the development of hepatorenal syndrome, hepatic encephalopathy and survival [5,6,7]. Such an important prognostic power has led serum sodium concentration to be included in the prognostic model for end-stage liver disease (MELD) [8], widely used to establish the need for liver transplantation (OLT) and prioritize patients on the waitlist, with the aim of improving its prognostic ability, especially in patients with cirrhosis and ascites. Namely, Biggins *et al.* [9] proposed the MELD-Na score by integrating serum sodium concentration into the MELD equation. This and a subsequent study [10], based on a larger sample of patients, suggested that MELD-Na score provides a better short-term mortality prediction among candidates for OLT than the original MELD score. It also emerged that the influence of hyponatremia was mainly evident with intermediate values of MELD score that can underestimate the severity of cirrhosis in specific settings, such as in patients with ascites. Further attempts to improve MELD score prognostic power are represented by the integrated MELD (iMELD), MELD to serum sodium ratio (MESO), and United Kingdom MELD (UKELD). The comparison between the performances of most MELD-based scores in waitlisted patients suggested that the most accurate scores to predict the drop-out rate from the waiting list are MELD-Na and iMELD. MELD-Na is the best drop-out predictor at three months, while both scores performed well at six months [11].

There are other reasons for the importance of hyponatremia in the liver transplant setting. Interestingly, the risk of waitlist mortality appears to increase by 12% for each unit of decrease in serum sodium concentration for values between 120 and 135 mmol/L [12]. Patients undergoing surgery with a reduced serum sodium concentration are at risk of developing irreversible neurological damage, such as central pontine myelinolysis, due to rapid correction of hyponatremia in the early postoperative period [13]. Moreover, they require a greater use of blood products and have a longer duration of hospital stay, as they are prone to develop neurological complications, renal failure, and bacterial infections during the first 30 days after transplant [14]. Lastly, patients with hyponatremia have an increased 3-month mortality with respect to patients without hyponatremia [14].

## 2. Pathophysiology

In healthy subjects, total body water balance and serum sodium concentration are maintained fairly steady, despite marked variations in daily fluid intake, by homeostatic mechanisms that induce changes in renal water handling. This response initiates within minutes, and consists of a complex interplay between baroreceptors, osmoreceptors and central neurohormonal systems located in the hypothalamus. The main effector factor of neurohormonal systems is represented by the antidiuretic hormone (arginine-vasopressin; AVP) that leads the epithelial cells of renal collecting tubules to modulate the expression of water-selective channels, known as aquaporins (AQP) [15].

AVP is synthesized in neurons of the supraoptic and paraventricular nuclei of the hypothalamus [16]. Its secretion is controlled by two separate pathways, which respond to different stimuli. The main pathway is represented by plasma osmolality. Indeed, in normal circumstances, plasma AVP concentration is closely and directly related to plasma osmolality, so that even tiny changes in the order of 1% (*i.e.*, 3 mOsm/kg) are associated with an average change in plasma AVP of 1 pg/mL, an amount sufficient to modify renal water excretion [17]. The afferent signals to the osmotic regulation of AVP synthesis and secretion are triggered by variations in intracellular water of osmoreceptors located in the anterior hypothalamus, close to the supraoptic nuclei, secondary to shifts in extracellular osmolality [18]. These changes occur through the expression of the mechanosensitive ion channels aquaporin 4 (AQP-4) [19].

**Figure 1:**
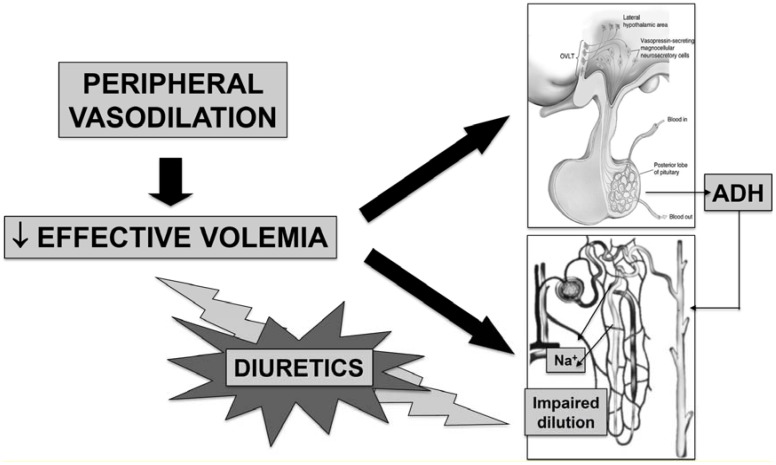
Main mechanisms impairing renal water handling and favoring dilutional hyponatremia in patients with advanced cirrhosis. Reduced effective volemia due to peripheral arterial vasodilation reduces renal perfusion, thus endangering free water generation (see also Figure 2), and enhances arginine-vasopressin secretion. In this context, the administration of diuretics and, mainly, loop diuretics, enhances both mechanisms, by reducing effective volemia and further impairing free water generation.

The other pathways regulating AVP secretion respond to nonosmotic stimuli involving the autonomic nervous system (parasympathetic pathways), which include changes in total or effective volemia [16]. In patients with cirrhosis, as occurs in other clinical conditions such as chronic heart failure, nephrotic syndrome, and hypothyroidism, the most frequent explanation for the AVP hypersecretion is a nonosmotic stimulation related to systemic hemodynamic abnormalities (Figure 1). In fact, advanced cirrhosis is characterized by a hyperdynamic circulatory syndrome that develops because of a reduction in systemic vascular resistance. This abnormality, which mainly involves the splanchnic circulatory area, ultimately leads to an even striking reduction of effective volemia that is not corrected by compensatory responses such as increased cardiac output and activation of vasoconstrictor systems [20]. The main pathophysiological mechanism leading to arterial vasodilation in cirrhosis is represented by an enhanced production of endothelium-derived vasodilating substances, among which nitric oxide (NO) plays a prevalent role [21]. Interestingly, the AVP biological effects are favored by NO, which plays a pivotal role in the regulation of arterial tone and, in particular, renal handling of sodium and water [22].

The systemic hemodynamic status of cirrhosis leads to a baroreceptor-mediated nonosmotic stimulation of AVP by unloading high-pressure baroreceptors, which explain why patients with cirrhosis can show sustained hyponatremia and hypo-osmolality to a degree that would suppress AVP release in normal subjects (Figure 1).

AVP is metabolized by the kidney and the liver [16], and a reduced liver clearance can be anticipated in patients with cirrhosis. This likely represents an additional factor leading to increased plasma concentration of the peptide.

The biological effects of AVP are mediated by three types of G protein-coupled receptors: V_1a_, V_1b_ and V_2_. V_1a_ receptors are responsible for vascular smooth muscle cell contraction, platelet aggregation and hepatic glycogenolysis; V_1b_ promotes adrenocorticotropin secretion by the anterior pituitary, and V_2_ receptors, located on the basolateral membrane of the principal cells of the collecting ducts, facilitate renal water reabsorption [15]. Namely, binding of AVP results in adenyl-cyclase activation, so that intracellular cAMP production is increased. This, in turn, activates a protein kinase (PKA) that promotes the migration and fusion of intracellular vesicles carrying the water channel AQP-2 to the luminal membrane of tubular epithelial cells. Membrane permeability to water is therefore enhanced. Water exits the cell thorough AQP-3 and -4, located in the basolateral membrane, drawn by the osmotic gradient generated by renal medulla. The maximization of this process leads to water reabsorption in excess of sodium, ultimately leading to dilutional hyponatremia [1,3].

**Figure 2:**
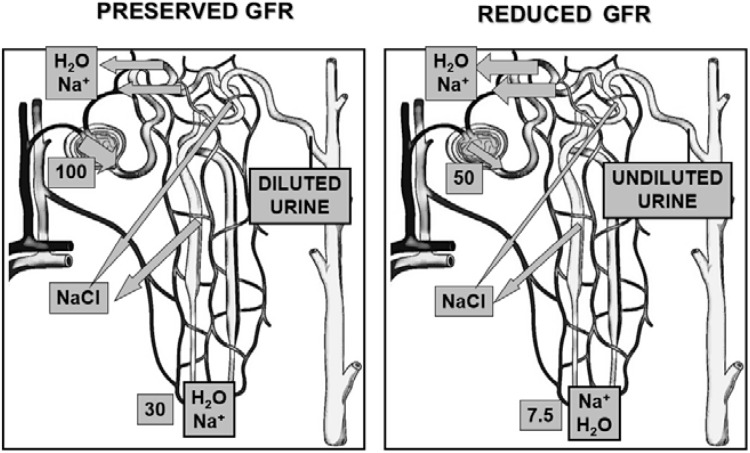
Schematic representation of sodium and water handling in case of preserved and reduced glomerular filtration rate (GFR). Preserved GFR: reabsorption of sodium and water in the proximal tubule is *iso*-osmotic and does not contribute directly to urine dilution, but determines the amount of fluid (sodium and water) delivered to the distal tubule. Set arbitrarily the filtered sodium and water load to 100, about 70% is absorbed by this segment, so that the amount of sodium delivered to loop of Henle is about 30. Tubular fluid is then diluted in the thick ascending limb of the loop of Henle, which is impermeable to water, due to the selective sodium reabsoption by the sodium-chloride-potassium symporter. Once diluted urine reaches the collecting duct, arginine-vasopressin suppression, which reduces permeability to water of the collecting duct, allows diuresis in excess of water. Thus, a water load can be eliminated and the ratio between water and solutes in the extracellular fluid restored. Reduced GFR: reduced filtration due to impaired renal perfusion lowers the filtered load of sodium and water (from 100 to 50) and enhances their proximal fractional reabsorption (about 85%). As a result, distal sodium delivery is severely impaired (7.5 *vs.* 30), endangering the efficacy of sodium reabsorpion in the ascending limb of Henle. An adequate urine dilution cannot ensue, so that a water load cannot be eliminated even though arginine-vasopressin secretion is suppressed. Dilutional hyponatremia then occurs. In addition to increased arginine-vasopressin secretion, this mechanism endangers renal water handling in patients with advanced cirrhosis.

Despite the AVP effects described above, the urine excretion of AQP-2 is reduced in patients with cirrhosis in parallel with the severity of cirrhosis. Indeed, the lowest levels are seen in patients with refractory ascites and hepatorenal syndrome [23], even though conflicting results have been reported in experimental studies [24,25]. The reasons for this finding are not entirely clear, but it would appear that they result from an adaptive renal response to sustained AVP hypersecretion, likely due to increased renal NO and/or prostaglandin synthesis [26], in order to avoid continuous renal solute-free water absorption potentially leading to lethal hyponatremia [23].

Although AVP undoubtedly plays a major pathophysiological role in the development of hyponatremia in cirrhosis, it has to be outlined that another pathogenetic mechanism is involved (Figure 2). Namely, patients with advanced cirrhosis and hyponatremia usually have an impairment of renal perfusion, leading to reduced glomerular filtration rate, as a result of effective hypovolemia and the compensatory activation of vasoconstrictor systems. In this condition, *iso*-osmotic sodium reabsorption at the proximal convoluted tubule is enhanced, thus leading to a striking reduction in distal nephron sodium delivery. Sodium reabsorption at the ascending loop of Henle is therefore blunted, and the urine dilution mechanism impaired [27]. Such a reduction of solute-free water clearance makes it impossible to eliminate a water load and is further affected by the administration of loop diuretics, whose mechanism of action consists in the inhibition of Na^+^-K^+^-2Cl symporter located in the thick ascending limb of the loop of Henle.

## 3. Clinical Features of Hyponatremia in Cirrhosis

### 3.1. Clinical Types

Patients with cirrhosis can develop two types of hyponatremia which differ markedly with respect to volume status: hypovolemic and hypervolemic hyponatremia.

Hypovolemic hyponatremia, which represents 10% of all hyponatremias in patients with cirrhosis [3], results from a substantial loss of extracellular fluid in excess of sodium, either from kidneys, as a result of high doses of diuretics, or the gastrointestinal tract due to diarrhea or vomiting. It is characterized by low serum sodium concentration associated with contraction of plasma volume, reduction in the total extracellular fluid volume with clinical signs of hypovolemia, such as tachycardia and reduced renal perfusion. While in patients without cirrhosis hypovolemic hyponatremia is characterized by the absence of edema, ascites and edema can coexist with severely reduced volemia in advanced cirrhosis. As arterial hypotension, tachycardia and renal failure can also result from reduced effective volemia secondary to hyperdynamic circulatory syndrome (see Pathophysiology), hypovolemic hyponatremia is not always easily recognizable in this setting.

In most cases, however, hyponatremia develops in the absence of major sodium losses in the context of expanded extracellular fluid volume with ascites and edema that results from renal fluid retention in excess of water with respect of sodium. In fact, although renal sodium retention is a cardinal feature of patients with advanced cirrhosis, solute-free water generation and, therefore, water excretion are also impaired to an extent that leads to a disproportionate increase in total body water relative to total sodium content, ultimately leading to dilutional hyponatremia.

This condition, known as hypervolemic hyponatremia, may occur spontaneously or as a consequence of excessive administration of hypotonic fluids or secondary to reduced renal perfusion, often precipitated by complications such as post-paracentesis circulatory dysfunction, hepatorenal syndrome and bacterial infections [28,29].

### 3.2. Clinical Manifestations

Hyponatremia is associated with a broad variety of neurological manifestations, whose intensity is related not only to the extent of serum sodium reduction, but also, and mainly, to the rate of fall. In fact, patients with acute hyponatremia have a much higher incidence of neurological symptoms than patients with chronic hyponatremia [3].

In patients without liver disease, the clinical effects of hyponatremia are related to brain edema, such as headache, disorientation, confusion, focal neurological deficits, seizures, and, in some cases, death due to cerebral herniation [30]. Moreover, hyponatremia leads to substantial changes in the brain intracellular environment to limit intracellular hyperhydration. These defense mechanisms consist of a rapid release of intracellular electrolytes, particularly potassium, which occurs within 24 h; subsequently, low-molecular-weight organic compounds, particularly myoinositol, are also discharged/released [30]. These changes require time to be reverted. Thus, a rapid increase in serum sodium concentration would overcome cell adaptation and brain shrinkage may ensue. This would trigger demyelination of pontine and extrapontine neurons that can cause neurologic dysfunction, including quadriplegia, pseudobulbar palsy, seizures, coma, and even death. Interestingly, in addition to malnutrition, potassium depletion, alcohol abuse, and hypocorticism, the risk of these complications is enhanced by liver cirrhosis [30].

In cirrhosis, hyponatremia generally develops slowly and gradually. Therefore, the brain can adjust to hypo-osmolality and hypotonicity of the extracellular fluid, so that the incidence of neurological manifestations directly attributable to hyponatremia is relatively low. However, since hyponatremia occurs in the setting of end-stage liver disease, it is often difficult to define to what extent the clinical manifestations are due to reduced serum sodium concentration or to hepatic encephalopathy. In fact, by favoring astrocyte swelling, hyponatremia becomes a major risk for the development of this complication, particularly in the settings of diuretic treatment, bacterial infections and transjugular intrahepatic porto-systemic shunts.

**Figure 3:**
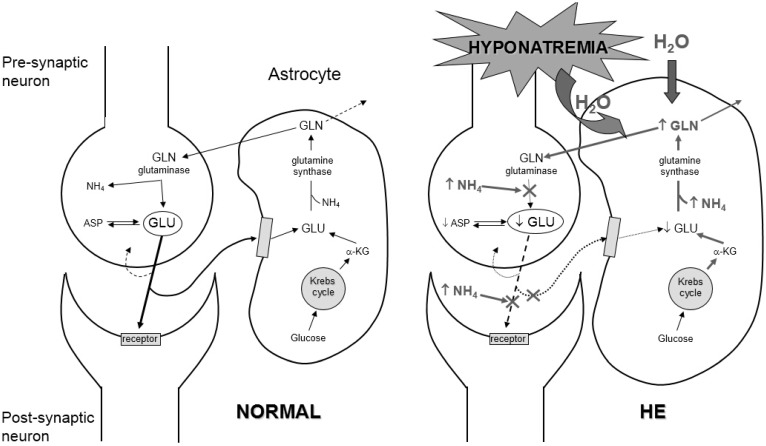
Schematic representation of glutamatergic pathway in normal subjects (NORMAL) and patients with hepatic encephalopathy (HE). Normal condition: α-ketoglutarate (α-KG), released by Krebs Cycle in astrocytes, is transformed to glutamate (GLU). The astrocytic glutamate pool is also supplied by its re-uptake from the synaptic cleft. Glutamate and ammonia (NH_4_) are converted into glutamine (GLN) by the enzyme glutamine synthase. Most glutamine moves into the pre-synaptic glutamatergic neuron, where the enzyme glutaminase re-converts glutamine into ammonia and glutamate. Upon excitation, the latter is released in the synaptic cleft, where it binds with the glutamate receptor located in the post-synaptic neuron, thus ensuring neurotransmission, is re-uptaken by the pre-synaptic neuron, and is uptaken by the astrocyte. Hepatic encephalopathy (HE): ammonia excess in the astrocytes enhances glutamine synthase activity: as a result, the glutamate and α-ketoglutarate pools are depleted, the latter impairing Krebs cycle activity. Excess glutamine enhances its release into the cerebrospinal fluid and transfer into the pre-synaptic neuron, where ammonia inhibits glutaminase activity. Neuronal glutamate pool is depleted and neurotransmission impaired. A further impairment of neurotransmission is due to excess ammonia in the synaptic cleft that interferes with glutamate binding to post-synaptic and astrocyte glutamate receptor. The increased astrocyte glutamine concentration osmotically recalls water from the extracellular fluid, ultimately leading to cell swelling. Hyponatremia favors water entry into astrocytes.

Hepatic encephalopathy is a neuropsychiatric syndrome that can occur in patients with advanced cirrhosis, portal hypertension and porto-systemic shunts. The pathophysiology of this complication is complex and is related to the effects of several “toxins”. These including beta-mercaptans, GABA, endogenous benzodiazepines *etc.*, but increased ammonia generation by the gut plays a major role [31]. Once ammonia has crossed the blood-brain barrier, it leads to an increased activity of the enzyme glutamine synthetase in astrocytes, which converts glutamate to glutamine. This mechanism, aimed at detoxifying ammonia, results in an intracellular accumulation of glutamine. The osmotic effect of this substance is responsible for intracellular hyperhydration and cell swelling (Figure 3). As a result, the intracellular concentration of osmotically active substances known as organic osmolytes and including myoinositol, which is the main organic osmolyte in human brain, choline, creatine, taurine and *N*-acetyl-aspartate is substantially reduced [31,32,33,34].

The extracellular fluid hypotonicity due to hyponatremia favors the osmotic effect of glutamine. Thus, cell swelling and cerebral edema induced by hyperammonemia are enhanced (Figure 3). Moreover, both hyperammonemia and hyponatremia alter myoinositol metabolism in brain cells. Thus, it can be easily understood that hyponatremia potentiates the neurological effects of altered ammonia metabolism [35] so that low serum sodium concentration and increased serum ammonia are major factors determining electroencephalographic abnormalities in cirrhosis [36]. Namely, the negative relationship between plasma ammonia concentration and mean dominant frequency is shifted towards the abscissa in parallel with the reduction in serum sodium concentration [36].

The liver transplant setting represents a condition at high risk for the occurrence of neurological complications, including central pontine myelinolysis, related to the rapid correction of hyponatremia. In a study of 347 adult patients, 3.5% presented severe hyponatremia (serum sodium concentration ≤127 mmol/L) at the time of surgery. Half of them developed neurological complications in the early post-operative period (central pontine myelinolysis in 3, convulsion in 2 and seizure in 1) [13]. The overall incidence of central pontine myelinolysis in a more recent study including 2175 primary OLT recipients was 0.5%, with a significant correlation with serum sodium level. Furthermore, although the serum sodium concentration at the time of OLT did not have a statistically significant impact on survival, patients with hyponatremia had more prolonged intensive care unit and hospital stay compared to normonatremic recipients [37].

## 4. Management of Hyponatremia in Cirrhosis

The distinction between hypovolemic and hypervolemic hyponatremia (see Clinical types) is very important for setting appropriate preventive measures and treatments.

### 4.1. Prevention

(1) Prevention of hypovolemic hyponatremia mainly consists in avoiding marked fluid losses in excess of sodium. The most frequent cause of hypovolemic hyponatremia in patients with cirrhosis and ascites is represented by diuretic overtreatment. Therefore, great care has to be paid in avoiding a markedly negative fluid balance. In practice, daily body weight reduction under diuretic treatment should not exceed 500–800 g [38]. Patients with peripheral edema appear to be protected from these effects because of the preferential mobilization of edema and may safely undergo diuresis at a more rapid rate (up to 2 kg/day) until edema disappears [38].
(2) In the majority of patients with advanced cirrhosis, hypervolemic hyponatremia develops in the setting of expanded extracellular fluid volume secondary to renal fluid retention in excess of water with respect to sodium. There are some measures that can help in preventing hypervolemic hyponatremia. First, it is inadvisable to administer hypotonic fluids to patient with ascites and impaired renal perfusion due to altered renal water metabolism. This includes the utilization of branched chain amino acids and glucose containing solution. Second, in case of complications that can acutely reduce effective volemia, there are established treatments aimed at preventing renal impairment and, therefore, dilutional hyponatremia. This is the case of post-paracentesis circulatory dysfunction (PPCD), which results from a further arterial vasodilation [39]. This complication may be effectively prevented with the administration of 8 g of human albumin/L of tapped ascites after the completion of large-volume paracentesis (>5 L) [29]. Indeed, this procedure not only prevents PPCD, but also its consequences, such hyponatremia and death [40]. Another condition that often induces an acute impairment of renal function, due to an infection-induced storm of pro-inflammatory cytokines, is represented by spontaneous bacterial peritonitis [41]. An albumin load (1.5 g/kg of body weight at diagnosis plus 1 g/kg b.w. on day three) in addition to antibiotic treatment significantly reduces the incidence of renal impairment and in-hospital and 3 month mortality [42]. Finally, treatment of hepatorenal syndrome with terlipressin and albumin also lead to an improvement of serum sodium concentration [43,44]. Indeed, terlipressin can induce hyponatremia because of V_2_ receptor-mediated water reabsorption in the collecting duct. However, this has been reported in patients with cirrhosis without renal failure receiving this drug because of variceal bleeding [45]. In hepatorenal syndrome the amelioration of effective volemia induced by V_1_ receptor stimulation by terlipressin overcomes its potential effects on V_2_ receptors [45].

### 4.2. Treatment (Figure 4)

(1) Hypovolemic hyponatremia cannot always be easily recognized in cirrhosis. Thus, the assessment of the clinical context where hyponatremia has ensued is crucial. The management consists of administration of normal saline and of identification and removal of the precipitating factor, which is often represented by diuretic overtreatment [3].
(2) The management of hypervolemic hyponatremia, persisting after the correction of possible precipitating events or apparently spontaneous, may be difficult.

**Figure 4:**
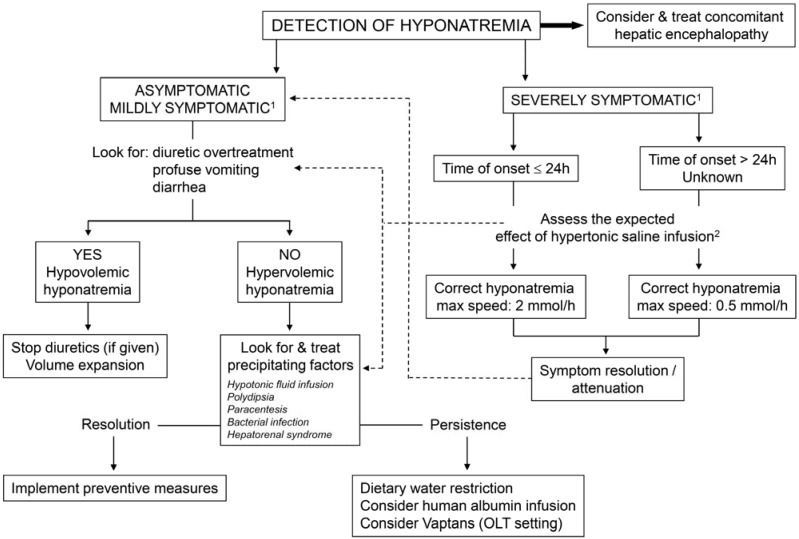
Proposed algorithm for the management of hyponatremia in patients with liver cirrhosis. ^1^ Severely symptomatic hyponatremia implies the presence of life-threatening manifestations, such as vomiting, cardio-respiratory distress, abnormal and deep somnolence, seizures and coma (Glasgow Coma Scale B8). Mild symptoms include nausea without vomiting, confusion, headache; ^2^ The expected effect of hypertonic saline infusion (and of any salt solution) can be calculated by the formula: (infused amount of Na^+^—actual Na^+^ serum concentration)/(total body water + 1) [30]; ^3^ Note that only Tolvaptan is available for clinical use. FDA (USA) has determined that this drug should not be used in patients with underlying liver disease because of the risk of severe liver injury.

Asymptomatic and mildly symptomatic hyponatremia, that is serum sodium concentration greater than 130 mmol/L, does not generally require a specific approach, even though there is no defined evidence as at what level of natremia treatment should start [3]. The key management of asymptomatic or mildly symptomatic hyponatremia would be to promote a negative water balance, with the aim of reducing total body water and, therefore, improving serum sodium concentration. Dietary water restriction is usually indicated [29], but is seldom effective. In fact, while fluid restriction is helpful in preventing a further decrease in serum sodium concentration, it is rarely effective in improving it. This lack of efficacy is likely due to the fact that, in practice, total daily fluid intake cannot be restricted to less than 1 L/day and the compliance of patients, who are often thirsty, is rarely achieved. In patients with persisting hyponatremia, reduction of diuretic dosage or at least temporary diuretic withdrawal have to be considered. Hypertonic sodium chloride administration, which can be indicated in severe, symptomatic hyponatremia (see below), has no role in this setting. Indeed, its efficacy is partial, usually short-lived, and increases the amount of ascites and edema. Albumin administration aimed at improving effective volemia might be effective, but data are very limited to recommend its use at present [29,46].

Severely symptomatic hyponatremia, as defined by the presence of life-threatening manifestations, such as vomiting, cardio-respiratory distress, abnormal and deep somnolence, seizures and coma (Glasgow Coma Scale B8) is not frequently seen in patients with cirrhosis. However, it always requires prompt and specific treatment, even though concomitant hepatic encephalopathy could contribute to these manifestations. In these cases hypertonic saline is indicated. As in other clinical settings [47], the initial rapid correction of hyponatremia should be guided by an improvement in clinical symptoms and the resolution of life-threatening manifestations, irrespective of the serum sodium concentration reached. Current clinical practice guidelines indicate that the infusion of hypertonic saline should be stopped once an improvement of symptoms after a 5 mmol/L increase in serum sodium concentration in the first hour has been achieved. Persisting symptoms would require continuing with the infusion, but at a lower rate (1 mmol/L/h) [47]. Unfortunately, the watershed beyond which correction of symptomatic hyponatremia has to be stopped or slowed can be scarcely definable in patients with advanced cirrhosis, where part of the symptoms may be ascribed to hepatic encephalopathy.

A fundamental requirement for the treatment of severe hyponatremia is that the entire deficit must not be corrected completely and rapidly, otherwise neurological sequelae, such as osmotic demylinisation, can be precipitated [30,47]. Thus, advisable correction rates should not exceed 12 mmol/L per 24 h (and <18 mmol/L per 48 h); the presence of additional risk factors for myelinolysis, which include advanced liver cirrhosis, would require even slower correction rates (<8–10 mmol/L per 24 h) [30,47].

The occurrence of seizures in patients with advanced cirrhosis, especially when a concomitant hepatic encephalopathy is present, merits discussion. While isolated attacks should not be treated if not with the partial correction of hyponatremia, the rare cases of status epilepticus may require pharmacological therapy. It should be pointed out, however, that hyponatremia-induced status epilepticus is often drug resistant [48] and, in any case, benzodiazepines should be avoided. In fact, benzodiazepines favor astrocyte swelling via peripheral benzodiazepine receptor, which is upregulated in hepatic encephalopathy [49]. Moreover, an increased GABAergic tone has been found in patients with hepatic encephalopathy [50]. GABA binding to its receptor results in inhibition of neurotransmission and a decrease in vigilance. Therefore, as benzodiazepine receptors are associated with GABA receptors, their administration may greatly amplify such an effect. Indeed, it has long been recognized that patients with cirrhosis are particularly sensitive to the administration of benzodiazepines [51].

Pharmacological treatment of hypervolemic hyponatremia could also be attempted. The use of demeclocyclin in patients with cirrhosis have been unsuccessful because of side effects [52]. The administration of low doses (0.5–1 mg/day) of the κ-opioid receptor agonist Niravoline, that inhibits antidiuretic hormone secretion, was able to induce water diuresis and increase serum sodium concentration. Higher doses, however, were associated with reversible personality disorders and mild confusion [53].

Selective blockade of the V_2_ receptors of AVP in the principal cells of the collecting ducts can be achieved by Vaptans. Indeed, these drugs are effective in improving serum sodium concentration in conditions associated with high vasopressin levels, such as the syndrome of inappropriate antidiuretic hormone secretion (SIADH) and heart failure [54]. The effects of the short-term administration (from one week to one month) of Vaptans to hyponatremic patients with cirrhosis and ascites have been assessed in several studies. Namely, it has been shown that Tolvaptan, Satavaptan and Lixivaptan lead to an increased urine volume and solute-free water excretion and improvement of hyponatremia in 45%–82% of cases [55,56,57]. In another study, a short intravenous infusion of Conivaptan for 1 to 4 days in patients with end stage liver disease awaiting OLT was also effective in increasing serum sodium concentration [58].

The most frequent side effect of aquaretics is thirst. Theoretical concerns related to their administration also include hypernatremia, dehydration, renal impairment, and osmotic demyelination syndrome due to a too rapid increase in serum sodium concentration, even though the incidence of such complications in the reported studies has been very low and no case of osmotic demyelination syndrome has been observed. In any case, treatment with these drugs must begin in a hospital setting with close clinical and laboratory monitoring in order to avoid increases of serum sodium of more than 8–10 mmol/L/day. Patients may be discharged after serum sodium concentration has been stabilized and no further increase in drug dose is required. Neither fluid restriction on the first day of therapy nor administration of saline should be used in combination with Vaptans to avoid a too rapid increase in serum sodium concentration. Treatment with these drugs may be considered in patients with severe hypervolemic hyponatremia (<125 mmol/L), especially in the pre-liver transplant setting.

The duration of treatment with Vaptans in patients with cirrhosis has not been established as yet. Safety has only been established for short-term treatment, up to one month. On this respect, phase III controlled clinical trials have been designed to test whether long-term Satavaptan administration in addition to diuretics may improve ascites in cirrhosis [59,60]. These studies showed that even though this drug was more effective than placebo in improving the serum sodium concentration in patients with hyponatremia, it did not significantly improve the control of ascites. Moreover, when Satavaptan was administered in combination with diuretics to prevent ascites recurrence after large-volume paracentesis, a higher rate of all-cause mortality, mostly associated with known complications of cirrhosis, was recorded during the 52 weeks of follow-up [60].

Finally, it should not be disregarded that Vaptans are metabolized by CYP3A enzymes in the liver and, therefore their metabolism could be impaired in patients with cirrhosis. Moreover, inhibitors, such as ketoconazole, grapefruit juice, and clarithromycin among others, or inducers, such as rifampin, barbiturates, and phenytoin, of the CYP3A system may substantially modify the effects of Vaptans.

It should be noted that, at present, only Tolvaptan has been approved for clinical use by FDA (USA) and EMA (Europe). The unique indication given by EMA is the syndrome of inappropriate antidiuretic hormone secretion (SIADH), while FDA also included heart failure and liver cirrhosis. However, the occurrence of serious hepatic injury in three patients with autosomal dominant polycystic kidney disease treated with Tolvaptan in a double-blind placebo-controlled trial [61] led FDA to determine that this drug should not be used in patients with underlying liver disease.

## 5. Conclusions

Hyponatremia is a common finding in advanced cirrhosis. Even though it is rarely severe enough to represent a life threatening condition, hyponatremia assumes an adverse prognostic meaning as it indicates an advanced disease with severe cardiovascular dysfunction. Indeed, hyponatremia in cirrhosis results from an impairment of effective volemia, mostly due to peripheral arterial vasodilation, leading to both non-osmotic, volume-driven AVP secretion and reduced renal perfusion and glomerular filtration rate that impair free-water clearance. The clinical manifestations of hyponatremia in cirrhosis do not differ from those seen in patients without cirrhosis. However, due to the concomitant abnormalities in nitrogen metabolism, symptoms amenable to hyponatremia are often associated with and hardly distinguishable from those related to hepatic encephalopathy. Treatment of hyponatremia in cirrhosis mainly relies on the defense of effective volemia. Precipitating factors have to be avoided or promptly recognized and corrected. Vaptans are undoubtedly effective in improving hyponatremia in cirrhosis. However, at present, their use is limited to the experimental setting.
